# Toothpick ingestion complicated by cecal perforation: case report and literature review

**DOI:** 10.1186/1749-7922-9-63

**Published:** 2014-12-19

**Authors:** Andrea Lovece, Emanuele Asti, Andrea Sironi, Luigi Bonavina

**Affiliations:** Department of Biomedical Sciences for Health, Division of General Surgery, IRCCS Policlinico San Donato, University of Milan Medical School, San Donato Milanese, Milano, Italy; Department of Surgery, IRCCS Policlinico San Donato, 20097 San Donato Milanese, Milano, Italy

**Keywords:** Toothpick, Ingestion, Perforation, Peritonitis

## Abstract

**Background:**

Diverticulitis and carcinoma represent the most common causes of colon perforation, but other causes, like ingestion of foreign bodies, should be taken into account.

**Case presentation:**

We report the case of a 64-year old man presenting in our Emergency Department with a 2 days history of right lower abdominal pain, nausea, vomiting and low grade fever. Physical examination evocated mild pain with positive rebound tenderness in the right lower abdominal quadrant, and positive right costovertebral angle tenderness. Routine blood tests, abdominal X-rays and CT scan were inconclusive for perforation. At explorative laparoscopy a cecal perforation with localized peritonitis was found, and a right colectomy was performed due to the suspicion of cancer. Histological examination confirmed the presence of a perforation caused by a piece of wood (toothpick) of 6 cm in length.

**Conclusions:**

Foreign body ingestion should be taken into account in the evaluation of acute abdominal pain. A detailed patient’s history may be crucial for a correct diagnosis and treatment.

## Background

Diverticulitis and carcinoma represent the most common causes of colon perforation. Other causes are iatrogenic perforation, penetrating abdominal trauma, and ingestion of foreign bodies. When animal bones, needles, toothpicks or other sharp pointed objects are ingested, the risk of perforation is even higher [1].

Toothpick ingestion is a relatively rare event that may results in serious gut injuries with peritonitis, sepsis or even death. Interestingly, a 4-year survey performed in the United States found 8176 reported toothpick-related injuries yearly, a rate of 3.6 per 100000 person-years [2]. We report a case of an unintentional ingestion of a toothpick causing cecal perforation.

## Case presentation

A 64-year-old man was referred to our emergency department with a two days history of right lower abdominal pain, nausea, vomiting and low grade fever. His vital signs were normal, except for a central body temperature of 38°C. There was no history of previous abdominal surgery. Physical examination evocated mild pain with positive rebound tenderness in the right lower abdominal quadrant, and positive right costovertebral angle tenderness. There was no evidence of intra-abdominal masses.

**Figure 1:**
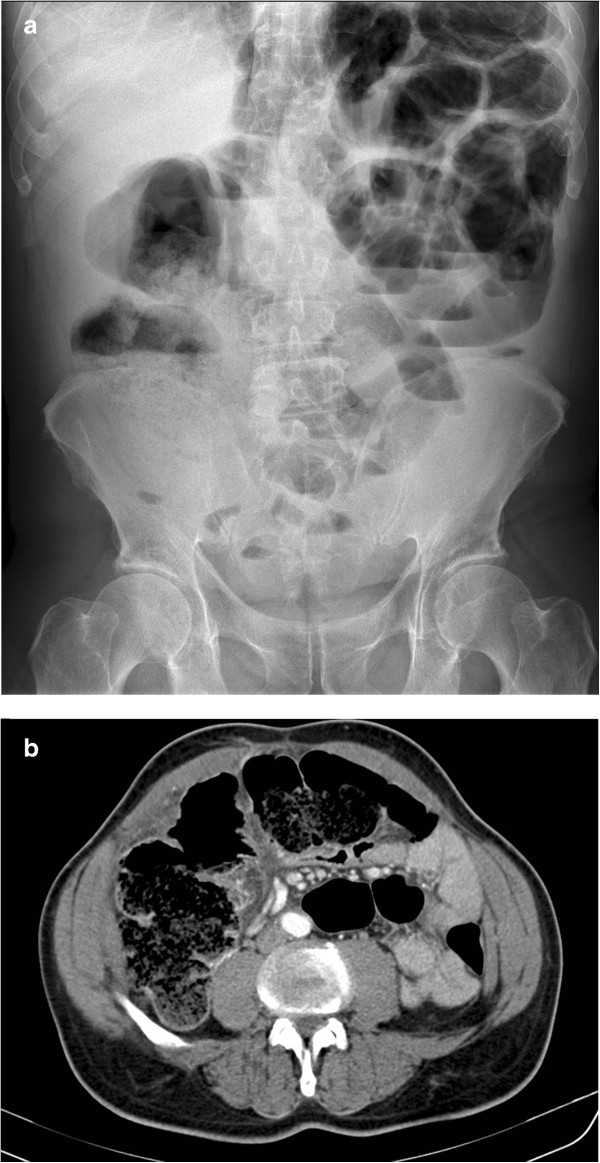
**a) X-ray of the abdomen (sagittal plane) showing distension of the small bowel with air fluid levels and no free air. b)** Contrast enhanced computed tomography (CT) slide (coronal plane). Extreme distension of the colon. No evidence of free air in the peritoneal cavity.

**Figure 2:**
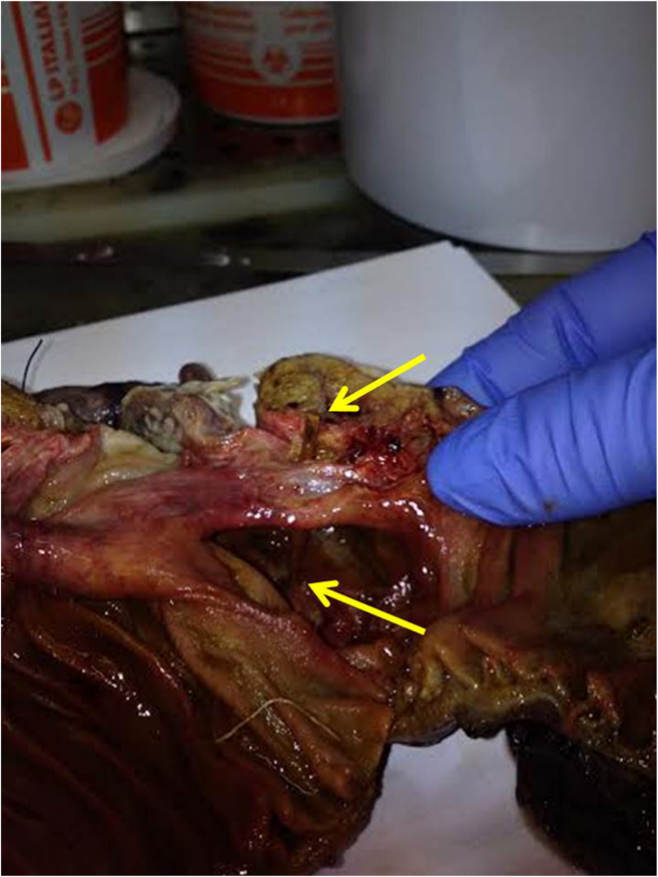
**Operative specimen showing the toothpick (arrows) and the site of transmural cecal perforation.**

Routine blood tests were normal except for a WBC count of 11,860/mm^3^ with 84,6% neutrophils. On plain abdominal X-rays the small bowel was distended and dislocated on the left upper abdominal quadrant with some air-fluid levels (Figure 1a). A contrast-enhanced abdominal computed tomography (CT) scan confirmed bowel occlusion with extreme ileum distension and about 50 ml of free fluid in the Douglas pouch; no evidence of pneumoperitoneum (Figure 1b). However, the cause of this obstruction remained unclear. The patient underwent explorative laparoscopy. A localized peritonitis around the cecum was found. The procedure was converted to a median laparotomy. A right colectomy with side-to-side semimechanical ileo-colic anastomosis was performed due to the suspicion of cancer. The patient made an uneventful recovery and was discharged on postoperative day 7^th^. The histological examination confirmed the presence of a transmural cecal perforation with abscess caused by a piece of wood (toothpick) of 6 cm in length (Figure 2). The patient admitted of having ate meat rolls as main course for dinner three days before hospital admission.

## Literature review

**Figure 3:**
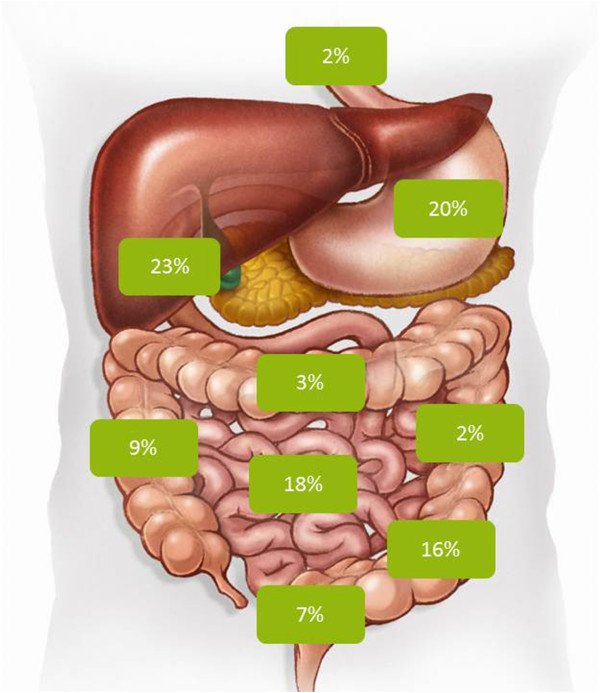
**Localization of toothpicks along the alimentary tract after ingestion.** Perforation occurred in 80% of the patients (from reference [3]).

A recent Medline literature research identified 136 case reports of ingested toothpicks [3]. Male gender, toothpick chewing habit, meals containing toothpicks and accompanied by alcoholic drinks were the main risk factors associated with toothpick ingestion. Most of the toothpicks were swallowed during meal time. Perforation of the alimentary tract occurred in 80% of these patients (Table 1); in one-third of them the toothpick had migrated into adjacent organs (liver, retroperitoneum, inferior vena cava, etc.). Figure 3 depicts the typical sites of toothpick lodgement along the alimentary canal. In 37% of the patients the toothpick had reached the colon [3]. Although the definitive identification of a toothpick on preoperative imaging is rare, a toothpick perforating the cecum after unconscious ingestion has been detected by ultrasound and CT scan in one patient [4].

**Table 1:** **Literature review of 136 patients with accidentally ingested toothpick (modified from reference 3)**

Male gender	74%
Mean age	52 (5–92)
Aware of ingestion	46%
Mean onset of symptoms	7 days
Perforation rate	79%
Sensitivity of diagnostic tests:	
- Ultrasound	32.6%
- Computed tomography	42.6%
- Endoscopy	72.1%
Therapy	Laparotomy (49%), endoscopy (30%), laparoscopy (9%)
Mortality rate	9.6%

## Discussion

Toothpick ingestion is a medical emergency since it leads to acute abdomen and gut perforation. In fact, most patients swallow toothpicks while eating sandwiches and are not able to recall or even unaware of this event. Since spontaneous elimination of these sharp foreign bodies through the gastrointestinal tract is unlikely, foreign body ingestion should be taken into account during the evaluation of acute abdominal pain.

The correct diagnosis is very difficult because of the low sensitivity of diagnostic investigations (Table 1). Endoscopy can be very helpful when the toothpick is localized in the upper-GI tract [5]. The CT scan may be able to identify the site of perforation and the extent of intra-abdominal inflammation either with or without abscess formation. Although the diagnostic yield is quite low, upper gastrointestinal endoscopy and ultrasound examination may be recommended in asymptomatic patients who are aware of the toothpick ingestion and seek medical advice within 24–48 hours [3].

Unfortunately, our patient was unaware of swallowing a wood toothpick that rapidly progressed and reached the colon. CT scan was unable to identify the cause of symptoms. Considering the presence of an acute abdomen, with evidence of peritonitis, the patient was scheduled for surgical exploration and required hemicolectomy.

This case suggests that a potential cause of lethal hazard may be unknown to the patient, but most of all it emphasizes the difficulty of a correct diagnosis with the most widely used instrumental exams, as endoscopy, ultrasound or CT scan.

## Consent

Consent to publish the case was obtained from our patient prior to discharge. The patient allowed us to share his story and clinical images because he understood the importance of raising awareness among physicians on this unusual surgical entity.

## Abbreviations

CT: Computerized tomography.
